# The effect of cesarean section on the dimensions and ratios of mons pubis

**DOI:** 10.1186/s12884-024-06667-w

**Published:** 2024-07-23

**Authors:** Mostafa Seleem, Omneya M. Osman, Sanaa G. Kashmar, Rehab Lotfy

**Affiliations:** Kasr Al-Ainy Maternity Hospital, Cairo University, Cairo, Egypt

**Keywords:** Mons pubis, Dimensions, Cesarean sections

## Abstract

**Background:**

Pregnancy and labor can impact women’s body contours. After a cesarean section, some women may experience aesthetic issues such as the formation of a panniculus and a bulging mons pubis. This study aimed to investigate the impact of cesarean sections on the dimensions of the mons pubis and their ratios.

**Methods:**

The study included 194 multiparous Caucasian women. Participants’ ages ranged from 18 to 40 years, and their BMI ranged from 18 to 30. They were divided into two BMI groups. Each group was further subdivided based on the mode of delivery into vaginal delivery (VD) and cesarean section (CS) groups. Manual measurements of the three dimensions of the mons pubis (monal height, monal width, and monal length) were conducted. Measurements were recorded in centimeters in the lithotomy position using iGaging 8-inch digital outside calipers. Monal height is the distance between the anterior surface of the symphysis pubis and the maximum height of the mons pubis (calculated by measuring the distance between the anterior wall of the vagina and the maximum height of the mons pubis minus the distance between the anterior wall of the vagina and the anterior surface of the symphysis pubis). Monal width is the maximum transverse distance between the merging borders of the mons pubis and the fat of the lower abdominal wall. Monal length is the maximum longitudinal distance between the merging upper border of the mons pubis and the fat of the lower abdominal wall and the upper end of the pudendal cleft.

**Results:**

No significant statistical difference was observed between the three dimensions of the mons pubis in vaginal delivery and cesarean section populations in the two groups. The changes in the ratios between the two groups’ different monal dimensions in the cesarean section population are minimal and do not follow a consistent pattern. There were no significant differences between the dimensions of single and repeated CS populations, with non-trendy changes in the different ratios in the repeated CS group.

**Conclusion:**

Even when repeated, cesarean section minimally affects the dimensions and ratios of the mons pubis. However, more studies with standardized fascial and subcutaneous fat closure are needed.

## Introduction

Pregnancy and labor can impact women’s body contours. During pregnancy, fat can accumulate in the lower abdomen, flanks, hips, thighs, and breasts [1]. Vaginal delivery and the pushing efforts during labor can weaken the abdominal wall, in addition to the weakness caused by the stretching of the abdomen during pregnancy. Lower segment cesarean section is the most commonly performed surgery, usually conducted through a lower transverse skin incision. For women, cesarean section is the most common repeated surgery [2].

Postnatally, some women may experience aesthetic issues with their abdominal wall. These issues include the formation of a panniculus and a bulging mons pubis [1]. The resulting scar from a cesarean section, particularly with repeat procedures, can affect the uniformity of subcutaneous fat and the continuity of Scarpa’s fascia [3]. It may prevent the fat of the mons pubis from expanding normally and maintaining its usual shape, especially if the scar is wide and indrawn. Additionally, the scar may interfere with lymphatic drainage, leading to lymph stasis and increased mons pubis size [4]. Therefore, our study aimed to investigate the impact of cesarean sections on the dimensions of the mons pubis and their ratios.

## Materials and methods

This cross-sectional study was conducted at Kasr Al-Ainy Obstetrics and Gynecology Hospital, Cairo University, from May to December 2021. The study included 194 multiparous Caucasian women seeking treatment for gynecological issues at Kasr Al-Ainy Hospital. Participants’ ages ranged from 18 to 40 years, and their BMI ranged from 18 to 30.

They were divided into two BMI groups: Group 1 consisted of 96 participants with a BMI ranging from 18.01 to 25, and Group 2 consisted of 98 participants with a BMI ranging from 25.01 to 30.

Each group was further subdivided based on the mode of delivery into vaginal delivery (VD) and cesarean section (CS) groups. The following groups of women were excluded:

- Pregnant women
- Women with vulvar disease or disorders
- Women with FGM (female genital mutilation)

After obtaining verbal and written consent from the participants, they underwent a thorough medical history assessment, general examination (including weight and height measurements), and BMI calculation. Finally, the vulva was examined, and measurements of the three dimensions of the mons pubis (monal height, monal width, and monal length) were recorded. The definitions of the different dimensions are as follows:

### Monal height (MH)

It is the distance between the symphysis pubis’s anterior surface and the mons pubis’ maximum height. It is calculated by measuring the distance between the anterior wall of the vagina and the maximum height of the mons pubis minus the distance between the anterior wall of the vagina and the anterior surface of the symphysis pubis.

### Monal width (MW)

It is the maximum transverse distance between the merging borders of the mons pubis and the fat of the lower abdominal wall.

### Monal length (ML)

It is the maximum longitudinal distance between the merging upper border of the mons pubis and the fat of the lower abdominal wall and the upper end of the pudendal cleft.

Measurements were recorded in centimeters in the lithotomy position, as it was the most convenient position for the women, using iGaging 8-inch digital outside calipers. To minimize variability, measurements were conducted by only one of the authors.

**Fig. 1:**
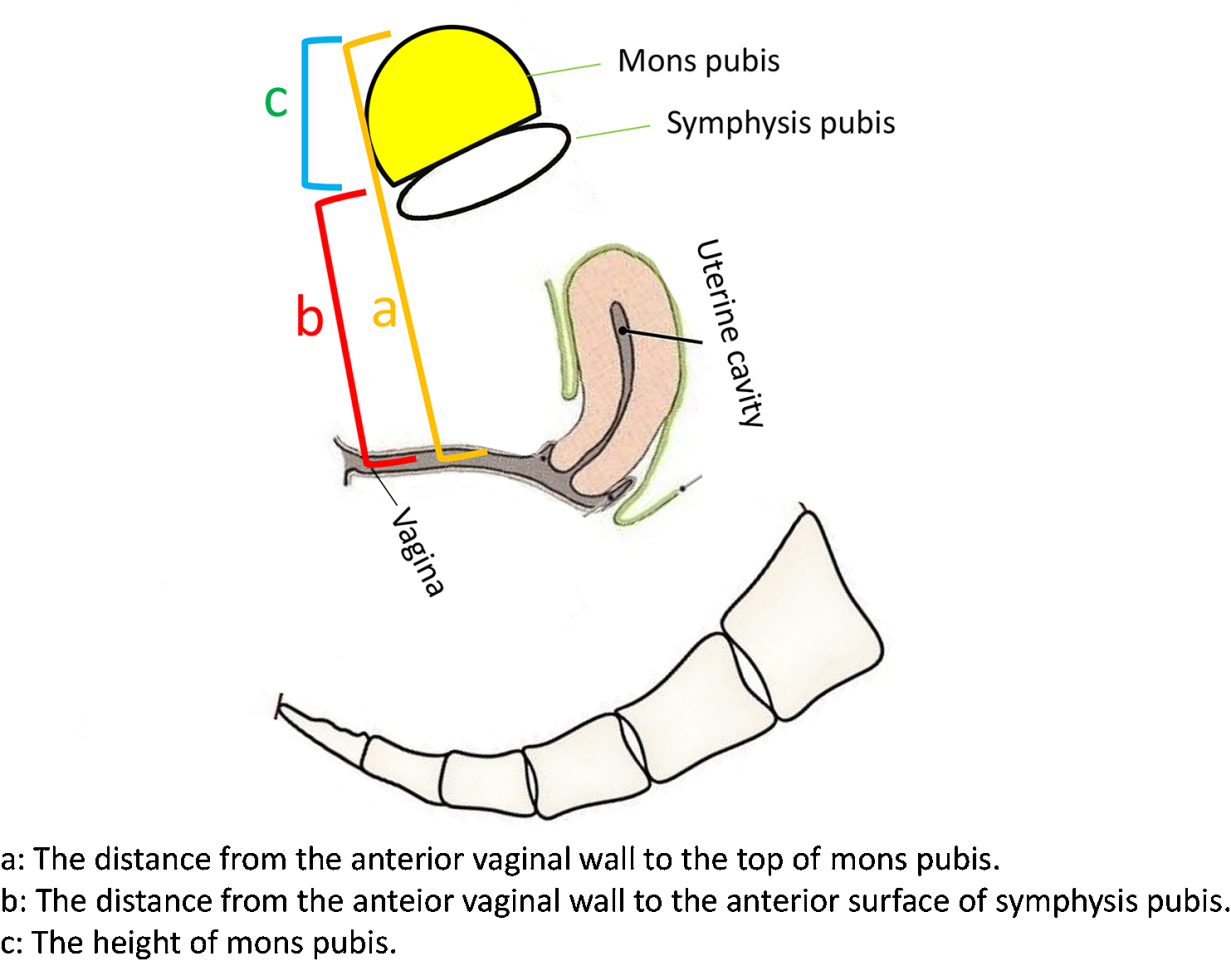
The two measured distances used to calculate the monal height

When measuring MH, one caliper of the vernier was positioned on the apex of the mons pubis. The other caliper was gently inserted into the vagina until both calipers were in the same sagittal plane, perpendicular to the coronal plane. The measurement was taken while applying slight pressure against the anterior vaginal wall. Following that, the upper caliper was placed on the anterior surface of the symphysis pubis, and another measurement was recorded. To ensure that both calipers were once again in the same sagittal plane) perpendicular to the coronal plane, ( the vaginal caliper had to be slightly withdrawn to align it with the upper caliper in the same sagittal plane (Fig. 1).

In measuring MW, the widely opened calipers of the vernier were laid flat below the umbilicus so that the calipers were lateral to the maximum width of the mons pubis. The calipers were then slowly brought closer together until they enclosed the lateral ends of the mons pubis at its widest points, where it begins to rise above the lateral skin levels.

When measuring monal length, one caliper of the widely opened calipers was initially placed at the inferior border of the mons pubis (the upper end of the pudendal cleft), and the remaining part of the tool was laid flat over the lower abdomen. The other caliper was gradually brought closer until it touched the point where the skin became elevated above the level of the lower abdomen skin.

After positioning and preparing the patient, three to five minutes were needed to complete all the measurements for each patient. After each examination, the measuring instrument was disinfected by submerging its calipers in CIDEX OPA solution for 20 min.

### Ethical considerations

This study was conducted after approval from the Research Scientific and Ethical Committee (RSEC) of the Department of Obstetrics and Gynecology, Faculty of Medicine, Cairo University (Approval Number: O210015).

### Statistical analysis

Data were entered into a computer and analyzed using IBM SPSS software package version 20.0 (Armonk, NY: IBM Corp). Quantitative data were described using range (minimum and maximum), mean, standard deviation, median, and interquartile range (IQR). The significance of the obtained results was judged at the 5% level. The student’s t-test was used to compare normally distributed quantitative variables between the two studied groups.

## Results

No statistically significant difference was detected between the mode of delivery and age in both groups (28.14 ± 4.02 vs. 27.85 ± 4.24 and 26.65 ± 4.22 vs. 27.49 ± 4.52 for VD and CS in Group 1 and Group 2, respectively).

The different measurements of the mons pubis in the two groups are shown in Table 1.

**Table 1:** Relation between mode of delivery and the taken measurements in Group 1 and 2

**Measurement**	**Group 1**			
	**VD** **(n = 49)**	**CS** **(n = 47)**	**Test of sig.**	**p**
MH	4.81 ± 0.88	5.15 ± 0.93	t = 1.857	0.066
ML	6.51 ± 0.6	6.53 ± 0.57	t = 0.181	0.857
MW	11.2 ± 1.23	11.64 ± 1.26	t = 1.705	0.092
**Measurement**	**Group 2**			
	**VD ** **(n = 51)**	**CS** **(n = 47)**	**Test of sig.**	**p**
MH	6.31 ± 1	6.26 ± 1.38	t = 0.241	0.801
ML	6.95 ± 0.9	7.16 ± 1.04	t = 1.064	0.290
MW	12.34 ± 1.6	12.3 ± 1.48	t = 0.145	0.885

MH: Monal height, MW: Monal width, ML: Monal length

Data was expressed in mean ± SD

t: Student t-test

p: p value for comparing between the two studied groups

In Group 1, a significant statistical difference in age was observed between single and repeated vaginal deliveries (25.91 ± 4.23 for single VD and 28.85 ± 3.73 for repeated VD) with a p-value of 0.03. At the same time, no significant statistical difference in age was observed between single and repeated cesarean Sect. (26.17 ± 4 for single CS and 28.43 ± 4.22 for repeated CS).

In Group 2, no significant statistical difference in age was detected between the number of deliveries in the VD population (25.5 ± 4.91 for single VD and 27.31 ± 3.85 for repeated VD). At the same time, a significant statistical difference was observed between the number of CS deliveries and age (25.56 ± 4.8 for single CS and 28.48 ± 4.1 for repeated CS) with a p-value of 0.034.

The comparisons between the measurements in single and repeated cesarean deliveries and vaginal deliveries in both groups are shown in Tables 2 and 3.

**Table 2:** Relation between the number of deliveries in CS and VD patients and the taken measurements in Group 1

**Measurement**		**CS group**								
		**1** **(n = 12)**		**> 1** **(n = 35)**		**Test of sig.**		**p**		
MH		5.38 ± 1.09		5.07 ± 0.87		t = 0.979		0.333		
ML		6.54 ± 0.58		6.53 ± 0.58		t = 0.067		0.947		
MW		11.42 ± 1.73		11.71 ± 1.08		t = 0.701		0.487		
		**VD group**								
**Measurement**		**1** **(n = 13)**		**> 1** **(n = 38)**		**Test of sig.**		**p**		
MH		5.14 ± 0.64		4.77 ± 0.99		t = 1.164		0.250		
ML		6.5 ± 0.67		6.5 ± 0.58		t = 0.0		1.0		
MW		10.86 ± 1.19		11.32 ± 1.23		t = 1.094		0.279		

MH: Monal height, MW: Monal width, ML: Monal length

Data was expressed in mean ± SD

t: Student t-test

p: p value for comparing between the two studied groups

**Table 3:** Relation between number of deliveries in CS and VD patients and the taken measurements in Group 2

**Measurement**		**CS group**								
		**1** **(n = 16)**		**> 1** **(n = 31)**		**Test of sig.**		**p**		
MH		6.28 ± 1.47		6.24 ± 1.36		t = 0.091		0.928		
ML		7.5 ± 0.75		6.98 ± 1.14		t = 1.636		0.109		
MW		12.56 ± 1.31		12.16 ± 1.57		t = 0.876		0.386		
**Measurement**		**VD group**								
		**1** **(n = 16)**		**> 1** **(n = 36)**		**Test of sig.**		**p**		
MH		6.53 ± 0.62		6.21 ± 1.11		t = 1.087		0.282		
ML		7 ± 0.66		6.93 ± 0.98		t = 0.258		0.797		
MW		12.56 ± 1.99		12.21 ± 1.41		t = 0.733		0.467		

MH: Monal height, MW: Monal width, ML: Monal length

Data was expressed in mean ± SD

t: Student t-test

p: p value for comparing between the two studied groups

Tables 4 and 5 show the different ratios and the percentage of change in the CS population in the two groups.

**Table 4:** The calculated ratios between the dimensions of the mons pubis and the percent of change in the CS population in the two groups

Ratio	Group 1			Group 2		
	VD	CS	%	VD	CS	%
**MH** to **MW**	0.43	0.44	+ 2%	0.51	0.51	0%
**MH** to **ML**	0.74	0.78	+ 5%	0.91	0.84	− 8%
**MW** to **ML**	1.72	1.78	+ 3%	1.78	1.72	-3%

MH: Monal height, MW: Monal width, ML: Monal length

-: Decrease

+: Increase

**Table 5:** The calculated ratios between the dimensions of the mons pubis in single and repeated cesarean section and vaginal delivery populations and the percent of change in repeated ones in the two groups

**Ratio**	**CS population**					
	**Group 1**			**Group 2**		
	**1** **(n = 12)**	**> 1** **(n = 35)**	%	**1** **(n = 16)**	**> 1** **(n = 31)**	%
**MH** to **MW**	0.47	0.43	− 8%	0.50	0.51	+ 2%
**MH** to **ML**	0.82	0.78	-5%	0.84	0.89	+ 5%
**MW** to **ML**	1.75	1.79	+2%	1.78	1.74	-2%
**Ratio**	**VD population**					
	**Group 1**			**Group 2**		
	**1** **(n = 13)**	**> 1** **(n = 38)**	%	**1** **(n = 16)**	**> 1** **(n = 36)**	%
**MH** to **MW**	0.47	0.42	-11%	0.52	0.51	− 2%
**MH** to **ML**	0.79	0.73	− 8%	0.90	0.90	0%
**MW** to **ML**	1.67	1.74	+4%	1.79	1.76	-2%

MH: Monal height, MW: Monal width, ML: Monal length

-: Decrease

+: Increase

## Discussion

The mons pubis is a pad of fat overlying the symphysis pubis and is continuous with abdominal wall fat above and the labia majora below. According to Seleem et al., the mons pubis is ellipsoid in shape with length, width, and height, and it is part of the fatty parts of the vulva [5].

The mons pubis is one of the body areas of women’s aesthetic concern [6]. Abdominal wall contour surgeries usually do not give enough attention to this body area. This lack of concomitant monsplasty may lead to disharmony and inadequate patient satisfaction [6].

Cesarean section may interrupt Scarpa’s fascia, subcutaneous fat, and rectus sheath continuity and harmony. Interruption of facial layers may interfere with sliding movement, lymphatic drainage, and the integrity of muscle function [3]. Non-closure of Scarpa’s fascia may lead to downward retraction of the fascia and subcutaneous fat [7]. Conversely, fibrosis and wound retraction may pull on the mons pubis, flattening it. As a result, the dimensions of the mons pubis may be altered in women with previous cesarean sections.

Our study is the first to shed light on the possible effects of cesarean sections on the aesthetics of the mons pubis. Our results showed no significant age difference between women who delivered vaginally and those who had cesarean sections in both groups. This indicates that any differences in dimensions are not due to age but rather the mode of delivery.

The results revealed no significant statistical difference in the three dimensions of the mons pubis between vaginal delivery and cesarean section populations in the two groups. For the vaginal delivery population, the measurements were as follows: MH (4.81 ± 0.88, 6.31 ± 1), ML (6.51 ± 0.6, 6.95 ± 0.9), and MW (11.2 ± 1.23, 12.34 ± 1.6) for Groups 1 and 2, respectively. For the cesarean section population, the measurements were: MH (5.15 ± 0.93, 6.26 ± 1.38), ML (6.53 ± 0.57, 7.16 ± 1.04), and MW (11.64 ± 1.26, 12.3 ± 1.48) for Groups 1 and 2, respectively.

It was observed that the changes in the ratios between the two groups’ different monal dimensions in the cesarean section population are minimal and do not follow a consistent trend. The changes range from + 2–0% for MH to MW, from + 5% to -8% for MH to ML, and from + 3% to -3% for MW to ML in Groups 1 and 2, respectively.

In vaginal delivery populations, we observed that in Groups 1 and 2, the dimensions in the population with multiple deliveries were not statistically significantly different from those in the population with only one vaginal delivery, showing slight, non-trendy changes in the ratios. These changes ranged from − 11% to -2% for MH to MW, from − 8 to 0% for MH to ML, and from + 4% to -2% for MW to ML in Groups 1 and 2, respectively. This suggests that the effect of pregnancy on dimensions and ratios and its relation to age should be further studied. Additionally, the combined effect of obesity (BMI over 30) and repeated delivery should be examined.

Furthermore, when comparing the dimensions of the mons pubis in populations with cesarean sections, we observed that in Groups 1 and 2, the dimensions in the population with repeated cesarean sections are not statistically significantly different from those in the group with only one cesarean section. The changes in ratios did not follow a consistent trend, ranging from − 8% to + 2% for MH to MW, from − 5% to + 5% for MH to ML, and from + 2% to -2% for MW to ML in Groups 1 and 2, respectively. This may be because repeated cesarean sections and the resulting scar can slightly push the fat and increase the height of the mons pubis, making it more prominent, or pull on and flatten the mons pubis. However, the changes in all ratios are minimal, within the range of 10%.

Although some women may complain that cesarean section may cause more bulging of the mons pubis, our results showed that even repeated cesarean sections do not significantly alter the dimensions of the mons pubis. The complaint of a bulging mons pubis after a cesarean section may be a false perception caused by the scar, which may be indrawn and create a division in the lower abdomen, giving women this false impression. It is important to note that our measurements were performed while women were in the lithotomy position, and the bulge may appear when standing due to skin and fascia laxity rather than increased fat deposition. Further studies on the effect of the standing position on the dimensions of the mons pubis may be necessary. This could change the management of these patients from monsplasty to monspexy.

Additionally, our results showed that the ratios of the different dimensions of the mons pubis did not follow a consistent pattern in the two groups and were not consistent with an increase in the number of deliveries or cesarean sections. This suggests that the effect of cesarean section on the dimensions and ratios of the mons pubis is minimal and variable. These inconsistent effects may depend on the site, width, and how much the scar is indrawn below the skin surface. Subcutaneous fat and Scarpa’s fascia closure may play a role in the effects on mons pubis dimensions.

In our study, we did not include women with a certain standardized subcutaneous closure technique, so the inconsistent changes in ratios may be attributed to that. Therefore, there is a need for a future study comparing the effect of cesarean section with or without subcutaneous closure on the dimensions of the mons pubis.

Furthermore, the possible variability in taking the different dimensions should be considered, so future studies with more participants are needed. Racial differences toward wound healing mandate conducting future studies with different racial backgrounds. Finally, the psychological impact of cesarean section on the mons pubis, as reported by women, should be studied in the future using scales like the Derriford Appearance Scale (DAS59) [8].

## Conclusion

Even when repeated, cesarean section minimally affects the dimensions and ratios of the mons pubis. However, more studies with standardized fascial and subcutaneous fat closure are needed to better understand and address potential impacts.

## Data Availability

No datasets were generated or analysed during the current study.

## References

[1] Matarasso A, Smith DM. Strategies for aesthetic reshaping of the postpartum patient. Plast Reconstr Surg. 2015;136(2):245–57. 10.1097/PRS.0000000000001410. 26218374 10.1097/PRS.0000000000001410

[2] Osayande I, Ogunyemi O, Gwacham-Anisiobi U, Olaniran A, Yaya S, Banke-Thomas A. Prevalence, indications, and complications of caesarean section in health facilities across Nigeria: a systematic review and meta-analysis. Reprod Health. 2023;20(1):1–21. 10.1186/s12978-023-01598-9. 10.1186/s12978-023-01598-9 PMC10237076 37268951

[3] Fan C, Guidolin D, Ragazzo S, et al. Effects of cesarean section and vaginal delivery on abdominal muscles and fasciae. Med. 2020;56(6):2–10. 10.3390/medicina56060260. 10.3390/medicina56060260 PMC7353893 32471194

[4] Tourani SS, Taylor GI, Ashton MW. Scarpa fascia preservation in abdominoplasty: does it preserve the lymphatics? Plast Reconstr Surg. 2015;136(2):258–62. 10.1097/PRS.0000000000001407. 26218375 10.1097/PRS.0000000000001407

[5] Seleem M, Osman OM, Kashmar SG, Lotfy R, Dimensions. Ratios, volumes, and weights of the fatty parts of the Vulva (Mons Pubis and Labia Majora). Aesthetic Surg J. 2023;43(9):1002–12. 10.1093/asj/sjad106. 10.1093/asj/sjad106 37067361

[6] Matarasso A, Wallach SG. Abdominal contour surgery: treating all aesthetic units, including the Mons Pubis. Aesthetic Surg J. 2001;21(2):111–9. 10.1067/maj.2001.114789. 10.1067/maj.2001.114789 19331881

[7] Husslein H, Gutschi M, Leipold H, Herbst C, Franz M, Worda C. Suture closure versus non-closure of subcutaneous fat and cosmetic outcome after cesarean section: a randomized controlled trial. PLoS ONE. 2014;9(12):1–12. 10.1371/journal.pone.0114730. 10.1371/journal.pone.0114730 PMC4262443 25494177

[8] Harris DL, Carr AT. The Derriford Appearance Scale (DAS59): a new psychometric scale for the evaluation of patients with disfigurements and aesthetic problems of appearance. Br J Plast Surg. 2001;54(3):216–22. 10.1054/bjps.2001.3559. 11254413 10.1054/bjps.2001.3559

